# Insulin receptor isoform A ameliorates long-term glucose intolerance in diabetic mice

**DOI:** 10.1242/dmm.025288

**Published:** 2016-11-01

**Authors:** Sabela Diaz-Castroverde, Almudena Gómez-Hernández, Silvia Fernández, Gema García-Gómez, Marianna Di Scala, Gloria González-Aseguinolaza, Elisa Fernández-Millán, Águeda González-Rodríguez, María García-Bravo, Pierre Chambon, Carmen Álvarez, Liliana Perdomo, Nuria Beneit, Oscar Escribano, Manuel Benito

**Affiliations:** 1 Department of Biochemistry and Molecular Biology II, School of Pharmacy, Complutense University of Madrid, Madrid 28040, Spain; 2 CIBER of Diabetes and Related Diseases (CIBERDEM), Health Institute Carlos III (ISCIII), Madrid 28029, Spain; 3 Mechanisms of Insulin Resistance Consortium (MOIR), Madrid 28040, Spain; 4 Division of Hepatology and Gene Therapy, Center for Applied Medical Research, University of Navarra, Pamplona, Navarra 31008, Spain; 5 Liver Research Unit, Hospital Universitario Santa Cristina, Instituto de Investigación Sanitaria Princesa, Amadeo Vives 2, Madrid 28009, Spain; 6 Centro de Investigación Biomédica en Red de Enfermedades Hepáticas y Digestivas (CIBERehd), Health Institute Carlos III (ISCIII), Madrid 28029, Spain; 7 Differentiation and Cytometry Unit, Hematopoietic Innovative Therapies Division, CIEMAT–CIBER of Rare Diseases (CIBERER)–Institute of Health Investigation Jiménez Díaz Foundation (IIS-FJD), Madrid 28040, Spain; 8 Institute of Genetic and Molecular and Cellular Biology (CNRS UMR7104; INSERM U596; ULP, Collége de France) and Mouse Clinical Institute, Illkirch, Strasbourg 67400, France

**Keywords:** Glucose homeostasis, Insulin receptor isoforms, Adeno-associated virus (AAVs), Liver

## Abstract

Type 2 diabetes mellitus is a complex metabolic disease and its pathogenesis involves abnormalities in both peripheral insulin action and insulin secretion. Previous *in vitro* data showed that insulin receptor isoform A, but not B, favours basal glucose uptake through its specific association with endogenous GLUT1/2 in murine hepatocytes and beta cells. With this background, we hypothesized that hepatic expression of insulin receptor isoform A in a mouse model of type 2 diabetes could potentially increase the glucose uptake of these cells, decreasing the hyperglycaemia and therefore ameliorating the diabetic phenotype. To assure this hypothesis, we have developed recombinant adeno-associated viral vectors expressing insulin receptor isoform A (IRA) or isoform B (IRB) under the control of a hepatocyte­-specific promoter. Our results demonstrate that in the long term, hepatic expression of IRA in diabetic mice is more efficient than IRB in ameliorating glucose intolerance. Consequently, it impairs the induction of compensatory mechanisms through beta cell hyperplasia and/or hypertrophy that finally lead to beta cell failure, reverting the diabetic phenotype in about 8 weeks. Our data suggest that long-term hepatic expression of IRA could be a promising therapeutic approach for the treatment of type 2 diabetes mellitus.

## Abstract (precis)

**Summary:** The specific hepatic expression of insulin receptor isoform A, but not isoform B, is able to revert, in the long term, the global glucose intolerance observed in diabetic mice.

## INTRODUCTION

Type 2 diabetes mellitus (T2DM) results from a combination of insulin resistance and impaired insulin secretion, where insulin resistance has been described as the most important pathophysiological feature in prediabetic states (Kahn, 1994, 1995). Insulin resistance affects several tissues that are relevant in glucose homeostasis such as liver, skeletal muscle and the adipose organ (Muoio and Newgard, 2008). In particular, hepatic insulin resistance is manifested by the blunted ability of insulin to activate its receptor kinase and its downstream targets, resulting in incomplete suppression of hepatic glucose production and therefore a clear hyperglycaemia. As a compensatory mechanism, beta cell hyperplasia and/or hypertrophy takes place to increase the insulin secretion that leads to hyperinsulinemia (Muoio and Newgard, 2008). Owing to the central role of the liver in the control of glucose homeostasis, hepatic insulin resistance becomes a hallmark of T2DM (Michael et al., 2000; Cook et al., 2015), and, therefore, precise regulation of glucose homeostasis is a major challenge in diabetes management (Callejas et al., 2003).

The insulin receptor (IR) is a member of subclass II of the tyrosine kinase receptor super-family that plays an essential role in glucose metabolism (Benyoucef et al., 2007; Ward et al., 2009). IR is closely related to other members of this family, like the insulin-like growth factor receptor (IGF-IR), which are involved in normal growth and development (Ward et al., 2009). In mammals, alternative splicing gives rise to two isoforms of IR; IRA and IRB. Isoform B has 12 additional amino acids encoded by exon 11 (Whittaker et al., 2009). This sequence is located immediately downstream of the ligand-binding domain but does not affect insulin binding affinity, which is very similar between IRA and IRB (Whittaker et al., 2009; Menting et al., 2013). However, IRA has ∼tenfold higher affinity for IGF-I and IGF-II than IRB (Malaguarnera and Belfiore, 2011). Moreover, IRA is predominantly expressed during foetal development and embryogenesis where it enhances IGF-II effects (Frasca et al., 1999). In addition to its role in development, it has also been described that an increased IRA/IRB ratio can be observed in beta cells under certain pathophysiological conditions such as insulin resistance. Therefore, these data suggest that the upregulation of IRA expression is not only involved in malignancies but also can be a compensatory mechanism by which beta cell mass can be expanded in response to a higher insulin demand (Escribano et al., 2009). Conversely, IRB is predominantly expressed in adult tissues, including the liver, where it triggers the metabolic effects of insulin (Malaguarnera and Belfiore, 2011). Although insulin does not stimulate glucose uptake in the liver, *in vitro* studies in neonatal hepatocytes and pancreatic beta cells demonstrate that IRA plays a direct role favouring glucose uptake, promoting its specific association with endogenous glucose transporters (GLUT1 and GLUT2) (Nevado et al., 2006; Escribano et al., 2015). Therefore, differences in the capability of glucose uptake can be associated with the presence or absence of IR isoforms or with changes in the ratio between them. Currently, the specific role of each IR isoform in T2DM is not completely understood.

To elucidate the role of IR isoforms, we took advantage of the capacity of recombinant adeno-associated vectors serotype 8 (AAV8) to deliver long-term expressed recombinant genes to the liver. These vectors have demonstrated an outstanding therapeutic potential in animal models for hepatic disorders of carbohydrate metabolism; for instance, correction of abnormal glycogen storage has been achieved (Alexander et al., 2008).

In the present study, we have used iLIRKO (inducible liver insulin receptor knockout) mice, as a model of severe hepatic insulin resistance and T2DM. iLIRKO present impaired glucose tolerance and fasted hyperinsulinemia without showing any hepatic dysfunction, a crucial difference with the previous reported LIRKO mice (Michael et al., 2000). We generated AAV8 vectors expressing IRA or IRB under the control of a hepatocyte-specific promoter, alpha-1 antitrypsin promoter (Kramer et al., 2003) in order to study the hepatic function of each insulin receptor isoform on the diabetic phenotype of iLIRKO mice. In this manuscript, we report that only specific hepatic IRA expression by AAV8 in iLIRKO mice is able to decrease the hyperglycaemia and plasma insulin levels, reverting the diabetic phenotype of these animals.

## RESULTS

### Generation and analysis of inducible liver insulin receptor knockout (iLIRKO) mice

**Fig. 1.**
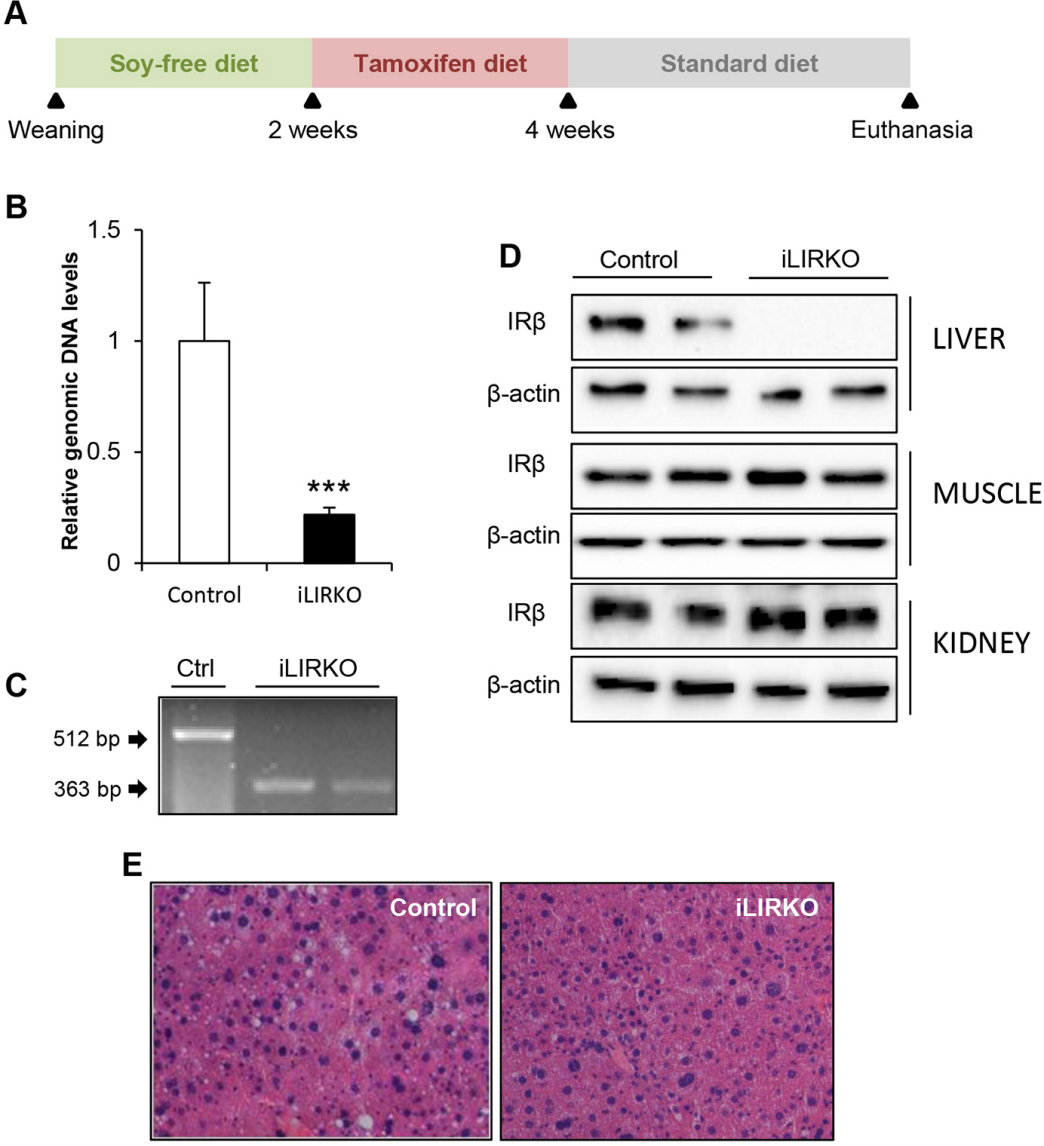
**Generation and characterization of iLIRKO mice.** (A) Schedule of diets for iLIRKO generation. (B) Administration of tamoxifen diet caused *Insr* exon 4 deletion in hepatocytes. Liver isolated genomic DNA from 5-month-old mice was analysed by qPCR. Data are means±s.e.m. for each experimental group (*n*=12). ***P<0.001 control versus iLIRKO by unpaired Student's *t*-test. (C) Mouse *Insr* exon 4 deletion was measured in livers from 5-month-old control and iLIRKO mice by RT-PCR. (D) Insulin receptor subunit beta (IRβ) expression was analysed by western blot in liver, muscle and kidney homogenates obtained from 5-month-old control and iLIRKO mice. β-actin was used as loading control. (E) H&E staining of random liver sections from 5-month-old control (left panel) and iLIRKO mice (right panel). Image magnification: 20×.

iLIRKO mice were created by breeding mice carrying insulin receptor alleles modified with loxP sites flanking exon 4 (Brüning et al., 1998) with mice in which the tamoxifen-dependent Cre-ER^T2^ recombinase gene was located under the albumin promoter (Schuler et al., 2004). After weaning, animals were fed with soy-free diet for two weeks in order to decrease phytoestrogen levels that could compete for binding to the Cre-ER^T2^ recombinase. Afterwards, mice were fed for two weeks with tamoxifen diet to induce Cre translocation to the nucleus and the deletion of *insulin receptor* (*Insr*) exon 4. Although glucose intolerance is associated with long-term administration of tamoxifen, for example in the treatment of breast cancer patients (Lipscombe et al., 2012), the animals were only fed with tamoxifen for 2 weeks, and glucose intolerance was not induced in control mice, demonstrating that the glucose intolerance in knockout mice did not result from the tamoxifen diet. Thereafter, animals were fed with standard chow until euthanasia (Fig. 1A). Deletion of *Insr* exon 4 was analysed by quantitative real-time PCR (qPCR) of genomic DNA samples isolated from the total pool of the liver from control or iLIRKO mice, showing that IR was sufficiently reduced in iLIRKO mice as compared with controls (Fig. 1B). The *Insr* exon 4 deletion was also tested by reverse transcription PCR (RT-PCR) in mRNA purified from the liver of control or iLIRKO mice (Fig. 1C), revealing the functionality of the exogenous inducible recombinase. Western blotting confirmed targeted specific disruption of IR in the liver of iLIRKO mice. No effect has been observed in other tissues such as muscle and kidney (Fig. 1D). Histological analysis at 5 months of age revealed normal hepatic architecture with no evidence of hyperproliferative nodules or steatosis in iLIRKO mice (Fig. 1E). There were no liver weight changes in iLIRKO mice as compared with control mice (data not shown).

### Insulin receptor deficiency in the liver causes marked glucose and insulin intolerance

**Fig. 2.**
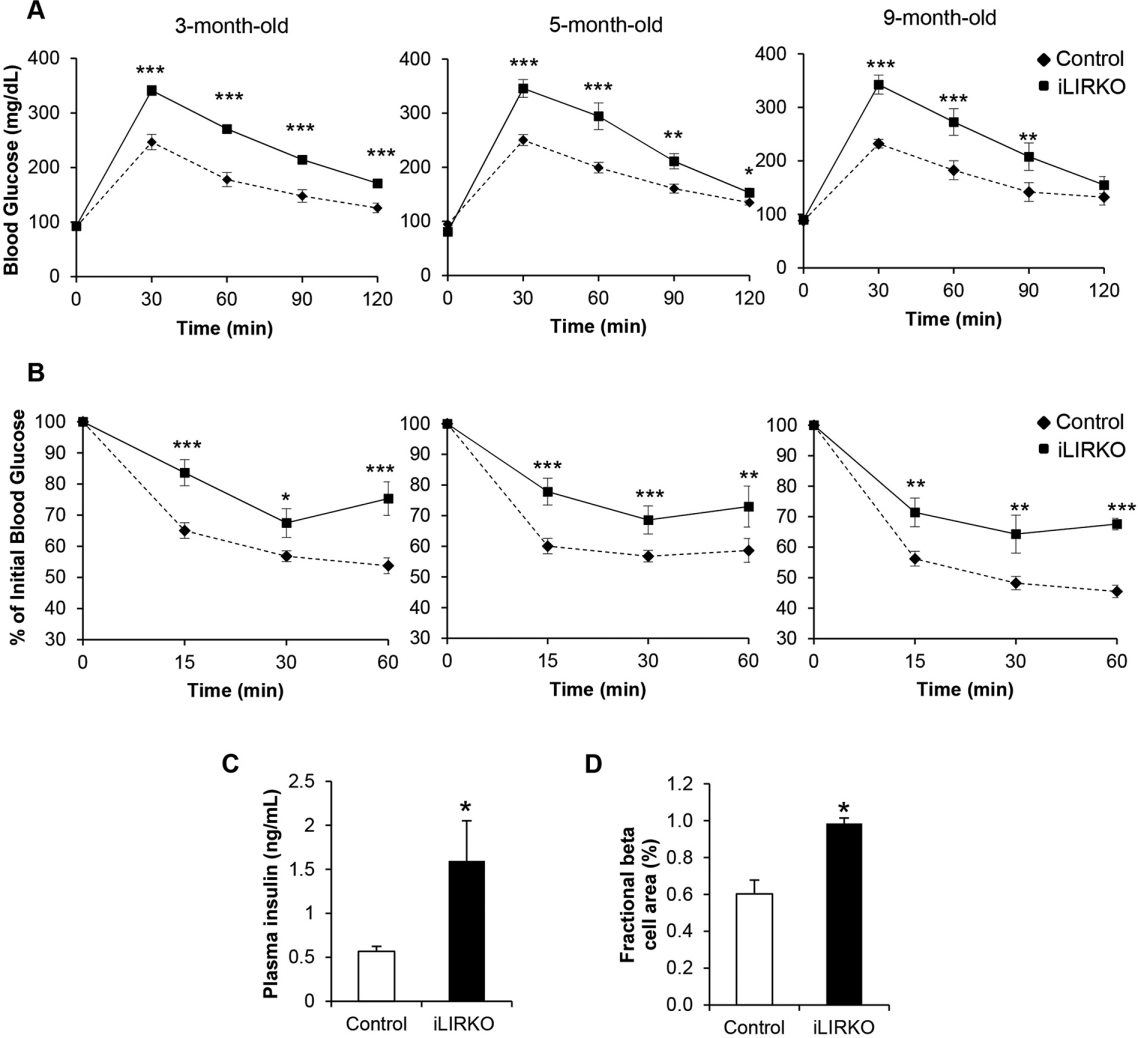
**Metabolic effects of hepatic insulin receptor ablation.** (A) Intraperitoneal glucose tolerance tests in 3-, 5- and 9-month-old control (diamonds) and iLIRKO (squares) mice (*n*=12 mice per group). (B) Intraperitoneal insulin tolerance tests in 3-, 5- and 9-month-old control (diamonds) and iLIRKO (squares) mice (*n*=12 mice per group). (C) Plasma insulin levels in 5-month-old control (open bars) and iLIRKO mice (filled bars) (*n*=12 mice per group). (D) Mean islet area in 9-month-old control (open bars) and iLIRKO mice (filled bars) (*n*=5 mice per group). Data are means±s.e.m. for each experimental group. *P<0.05, **P<0.005, ***P<0.001 control versus iLIRKO mice by one-way ANOVA with Bonferroni post test in A,B and unpaired Student's *t*-test in C,D.

Glucose and insulin tolerance were significantly impaired in 3-month-old iLIRKO mice, and both effects were fully maintained up to 9 months (Fig. 2A,B). These mice developed a marked hyperinsulinemia, resulting from the compensatory insulin secretion from pancreatic beta cells (Fig. 2C). We next characterized beta cell mass in control and iLIRKO mice. In accordance with the hyperinsulinemia, insulin staining revealed a significant increase in beta cell mass of ∼twofold in iLIRKO versus control mice at 9 months of age (Fig. 2D). Consistent with previous works, these results indicate that hepatic IR signalling is required for normal glucose homeostasis.

### AAV-mediated IRA/B expression in the liver

**Fig. 3.**
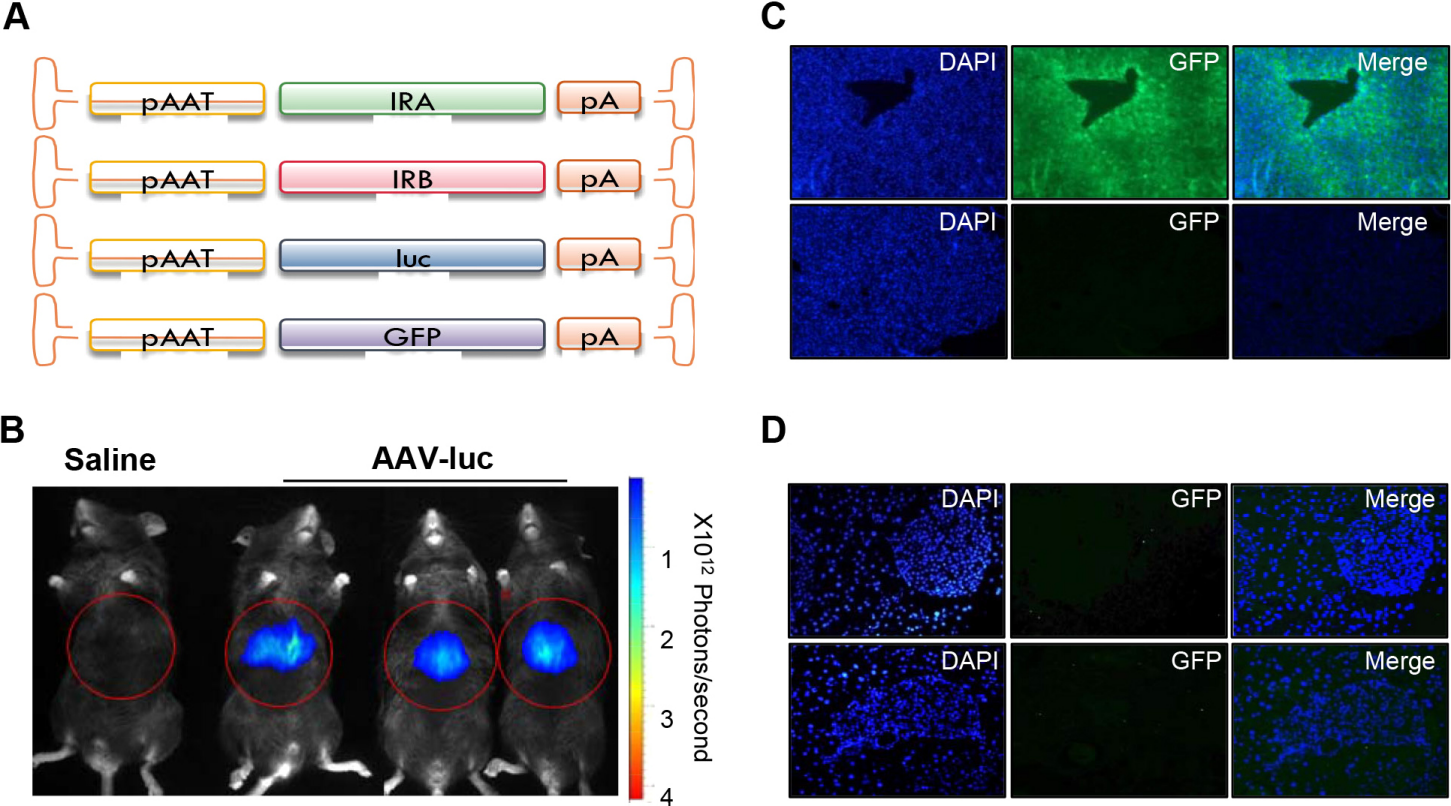
***In vivo* characterization of AAV-GFP- and AAV-luc-injected mice.** (A) Schematic diagram of adeno-associated viral (AAV) vectors used in this study. pAAT, human *α1-antitrypsin* promoter; GFP, *green fluorescent protein* gene; IRA/B, *INSR* A or B isoform; ITR, inverted terminal repeat; *luc*, *luciferase* gene; polyA, polyadenylation signals. (B) C57BL/6 mice intravenously injected with saline solution (left) or 3×10^10^ vg/kg of AAV-luc (right) were analysed by *in vivo* luciferase imaging 21 days after vector injection with a charge-coupled device (CCD) camera. Optical CCD images for luciferase expression of a representative animal from each group are shown. Liver area is circled in red. (C) Representative immunofluorescences show GFP expression in the liver and (D) pancreas from AAV-GFP-injected mice 15 days after injection with 3×10^10^ vg/kg of AAV-GFP (upper panels) and negative control staining of GFP (lower panels). Image magnification: 10× in C, 20× in D.

To explore the importance of each IR isoform in the liver on the normalization of global glucose homeostasis, we generated recombinant adeno-associated virus serotype 8 vectors containing human genes encoding IRA (AAV-IRA) or IRB (AAV-IRB) isoforms, or the reporter genes luciferase (AAV-luc) or green fluorescent protein (AAV-GFP) under the control of a liver-specific promoter (Fig. 3A). Liver specificity was demonstrated by *in vivo* luciferase expression analysis comparing saline-injected versus AAV-luc-injected mice (Fig. 3B). To test the percentage of transduced hepatocytes, we injected 3×10^10^ vector genomes (vg)/kg AAV-GFP into control mice. The results show that ∼95% of hepatocytes were GFP-positive within two weeks of treatment, suggesting that this dose led to a robust hepatic transduction (Fig. 3C). Lower panels represent GFP staining of the negative control. We also analysed GFP staining in pancreas from AAV-GFP-injected mice to confirm the AAV8 virus hepatic tropism (Fig. 3D). As shown, no GFP staining was observed in the pancreas of these animals. These results rule out the possibility of IR isoform expression within the pancreas owing to AAV8 incorporation in this tissue.

**Fig. 4.**
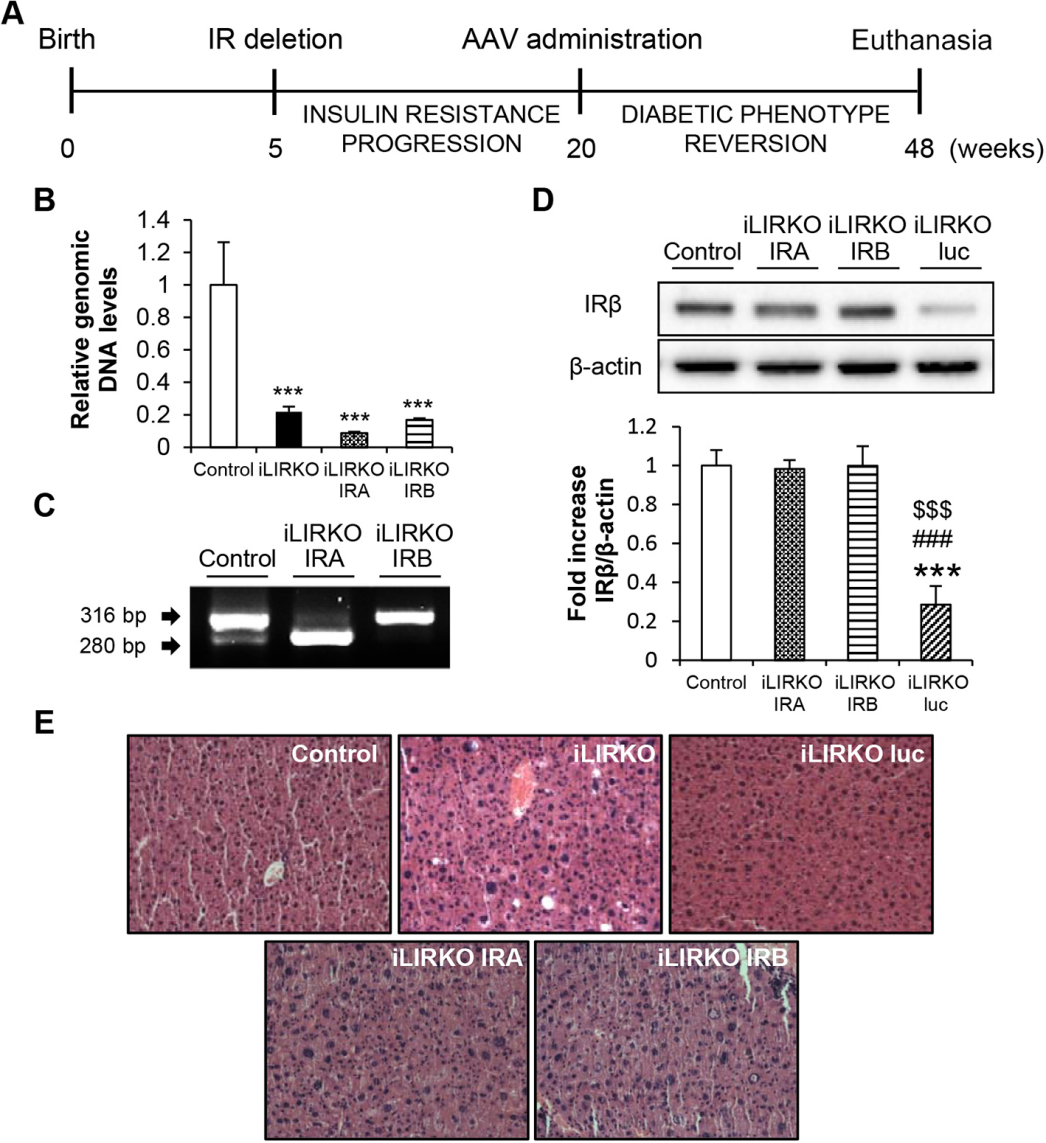
**AAV-mediated IRA and IRB expression in the liver recovered initial levels of insulin receptor.** (A) Schedule of AAV administration experiments. (B) Mouse genomic DNA *Insr* exon 4 levels were analysed by qPCR in 9-month-old control and iLIRKO mice with or without AAV-IRA or AAV-IRB administration. (C) Mouse *Insr* isoforms in control and human *INSR* isoforms in iLIRKO IRA and iLIRKO IRB were analysed by RT-PCR in livers from 9-month-old mice. (D) Representative IRβ expression analysed by western blot in liver homogenates from 9-month-old control, iLIRKO IRA, iLIRKO IRB and iLIRKO luc mice. β-actin was used as loading control. The histogram shows the band intensity quantification. Data are means±s.e.m. for each experimental group, *n*=5. (E) Representative H&E staining of liver sections from 9-month-old control, iLIRKO, iLIRKO luc (upper panels) and iLIRKO IRA, iLIRKO IRB (lower panels) mice. Image magnification: 20×. ***P<0.001 versus control mice; ^###^*P*<0.001 versus iLIRKO IRA; ^$$$^*P*<0.001 versus iLIRKO IRB by one-way ANOVA with Bonferroni post test.

To confirm whether the IR isoforms have a differential role in the reversion of the diabetic phenotype of iLIRKO mice we designed the experimental schedule shown in Fig. 4A. AAV vectors were administrated 20 weeks after birth, when iLIRKO mice achieved the diabetic phenotype, and then we checked the evolution of glucose and insulin tolerance at different time points. Mice were euthanised 28 weeks after treatment (Fig. 4A). Genomic levels of mouse *Insr* exon 4 were measured by qPCR in all the groups studied. The results showed similar mouse exon 4 deletion in liver homogenates from AAV-IRA-injected iLIRKO (iLIRKO IRA) and AAV-IRB-injected iLIRKO (iLIRKO IRB) compared with iLIRKO mice, indicating that all the IR expression observed after the AAV administration was coming from the AAV vectors carrying the human IR isoforms transgenes (Fig. 4B). After AAV administration, mice achieved the expression of the corresponding *INSR* isoform in the liver as analysed by RT-PCR (Fig. 4C). Following determination of AAV-mediated IR expression in the liver, we examined protein levels by western blot. Twenty-eight weeks post-vector injection, IRA or IRB expression was similar to that observed in control mice, avoiding any side effects related to IR overexpression. There was no effect on protein expression in iLIRKO mice injected with AAV-luc (Fig. 4D). Even though IRA overexpression is associated with hepatocellular carcinoma, it is noteworthy to emphasize that there were no alterations in hepatic morphology or structure in either iLIRKO-IRA or iLIRKO-IRB mice even after 28 weeks of the AAV administration as assessed by Dr Fernández-Aceñero, pathologist from San Carlos Clinic Hospital in Madrid (Spain). The quantitative pathology score obtained was similar in the five groups studied, revealing no liver alterations resulting from gene manipulation (Fig. 4E). Thus, our data indicate that long-term AAV-mediated expression of IRA or IRB meets safety requirements for a gene therapy approach.

### IRA expression in the liver improves glucose homeostasis

**Fig. 5.**
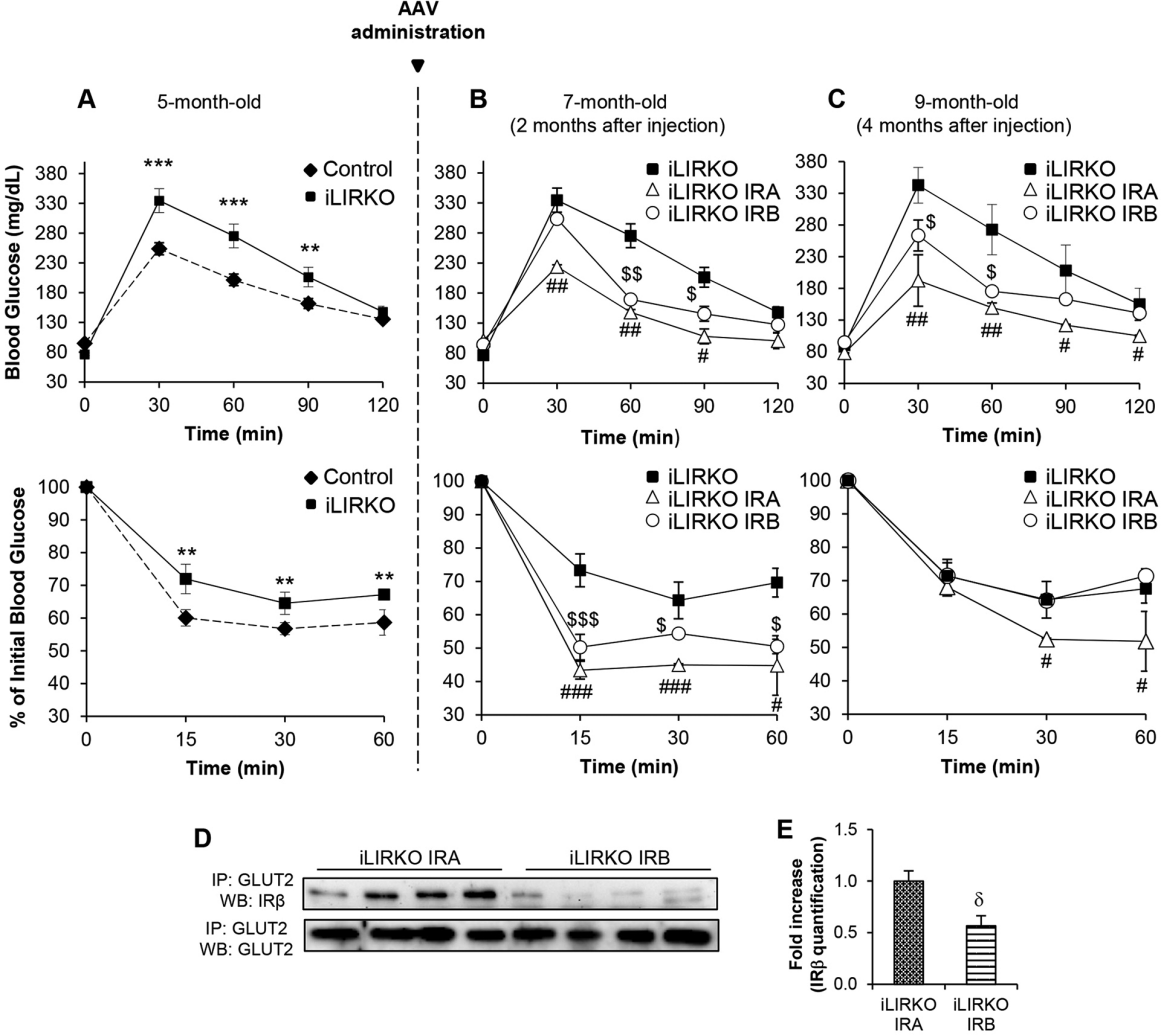
***In vivo* IRA,** **but not IRB,** **expression in hepatocytes reverts insulin resistance and glucose intolerance.** (A) Intraperitoneal glucose (upper panel) and insulin (lower panel) tolerance tests in 5-month-old control (diamonds, *n*=15) and iLIRKO mice (squares, *n*=10). (B,C) AAV-IRA or AAV-IRB were administrated in these iLIRKO mice (*n*=5 per isoform). GTT and ITT in 7- and 9-month-old iLIRKO (*n*=12), iLIRKO IRA (white triangles, *n*=5) and iLIRKO IRB (white circles, *n*=5). (D) Association between GLUT2 and IRβ in liver homogenates from 9-month-old iLIRKO IRA (*n*=4) and iLIRKO IRB (*n*=4) mice. Samples were subjected to immunoprecipitation (IP) and western blot (WB). (E) Histogram of band intensity quantification. (A-C,E) Data are means±s.e.m. for each experimental group. **P<0.005, ***P<0.001 control versus iLIRKO; ^#^*P*<0.05, ^##^*P*<0.005, ^###^*P*<0.001 iLIRKO versus iLIRKO IRA; ^$^*P*<0.01, ^$$^*P*<0.005, ^$$$^*P*<0.001 iLIRKO versus iLIRKO IRB; ^δ^*P*<0.01 iLIRKO IRA versus iLIRKO IRB by one-way ANOVA with Bonferroni post test in A-C, unpaired Student's *t*-test in E.

We next tested the effects of IRA or IRB hepatic expression on glucose homeostasis by performing glucose and insulin tolerance tests in iLIRKO mice just before vector injection, and 2 and 4 months after vector injection (Fig. 5A-C). Treatment with both vectors improved glucose and insulin tolerance. However, as shown in Fig. 5B, it took 2 months from injection of IRA, but not IRB, to restore blood glucose to the levels found in 5-month-old control animals (Fig. 5A). Moreover, these differences between iLIRKO IRA and iLIRKO IRB remained for 4 months after viral administration (Fig. 5C). These results suggest that IRA is more efficient in the long term than IRB in restoring global glucose homeostasis in iLIRKO mice. In this sense, we have evaluated several parameters regarding glucose and lipid metabolism. The most remarkable result is the significant increase in hepatic glycogen content observed in iLIRKO IRA mice compared with the other groups studied (Table S1). To investigate potential involvement of an underlying molecular mechanism, we performed immunoprecipitation assays searching the possible *in vivo* association between IR isoforms and GLUT2 (Fig. 5D,E). The results obtained show a significantly higher association between IRA and GLUT2 than those observed between IRB and GLUT2 in the corresponding iLIRKO mice.

### IRA, but not IRB, expression in the liver induces a decreased beta cell mass

**Fig. 6.**
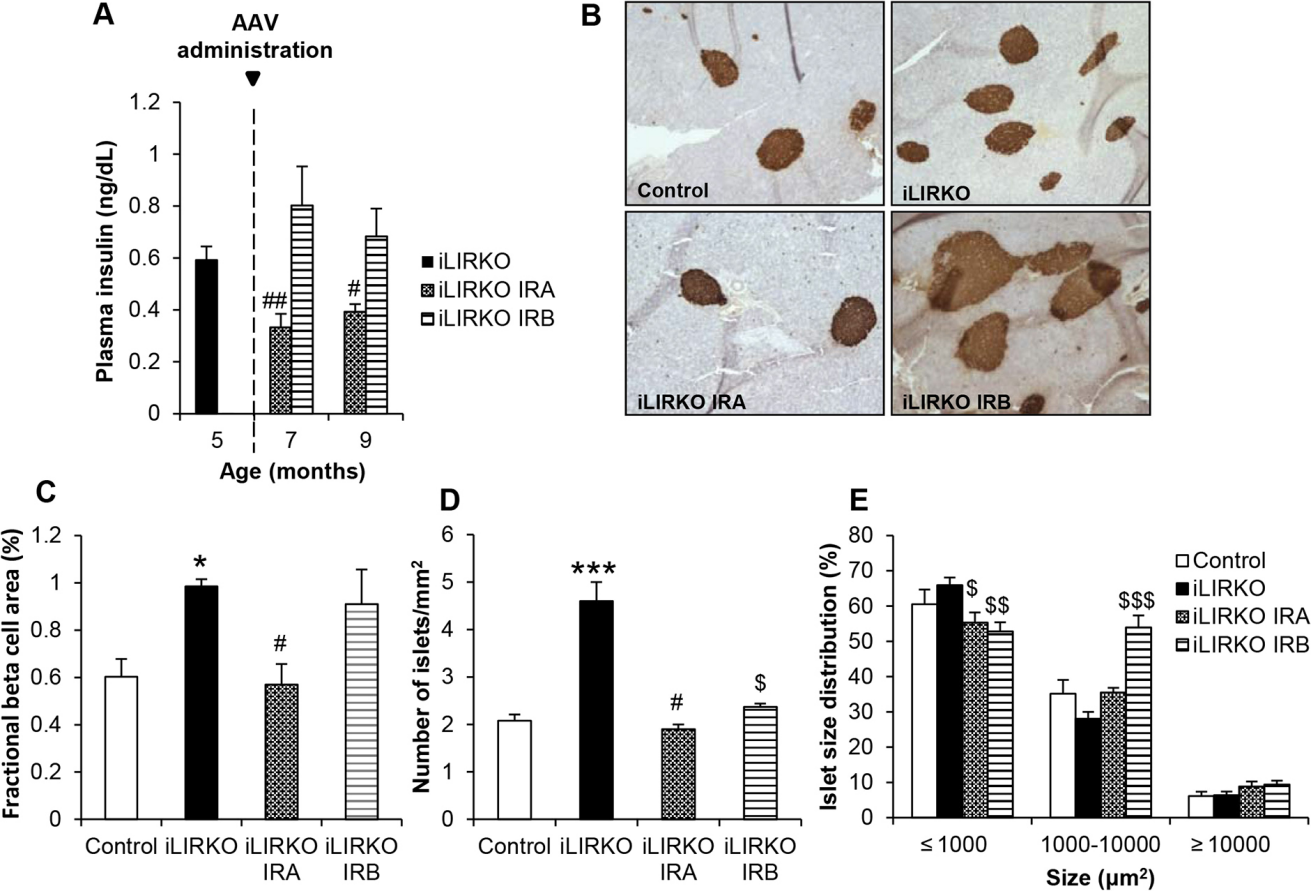
***In vivo* expression of IRA in hepatocytes reverts compensatory beta cell hyperplasia.** (A) Plasma insulin levels in 16-h-fasted 9-month-old mice. Data are means±s.e.m. for each experimental group (*n*=12). ^#^*P*<0.01, ^##^*P*<0.005 iLIRKO versus iLIRKO IRA by unpaired Student's *t*-test. (B) Representative insulin staining of pancreatic sections from 9-month-old mice of the four groups studied. Image magnification: 10×. (C-E) Fractional beta cell area (C), Islet density (D) and Islet size distribution (E) of control, iLIRKO, iLIRKO IRA and iLIRKO IRB mice. Data are means±s.e.m. for each experimental group (*n*=5). *P<0.05, ***P<0.001 control versus iLIRKO; ^#^*P*<0.05 iLIRKO versus iLIRKO IRA; ^$^*P*<0.05, ^$$^*P*<0.005, ^$$$^*P*<0.001 iLIRKO versus iLIRKO IRB by unpaired Student's *t*-test.

Examination of beta cell mass in iLIRKO IRA or iLIRKO IRB mice revealed that the improvement of hyperglycaemia induced by hepatic IRA expression has a direct effect on beta cell plasticity. We found that plasma insulin levels were significantly decreased in iLIRKO IRA mice at 2 or 4 months upon injection as compared with the same iLIRKO IRA mice before injection. By contrast, in iLIRKO IRB mice, plasma insulin levels remained elevated at 2 or 4 months upon AAV administration (Fig. 6A). As shown in a representative insulin staining (Fig. 6B), quantification of fractional beta cell mass revealed an increase in iLIRKO mice compared with control mice, owing to the lack of insulin receptor. Regarding IR isoforms, our results demonstrated a significant decrease in beta cell mass in iLIRKO IRA mice, but not in iLIRKO IRB mice, as compared with iLIRKO (Fig. 6C). These results imply that iLIRKO mice experience an increase in the number of islets as compared with control mice. However, we found a significant decrease in the number of islets in both iLIRKO IRA and iLIRKO IRB mice, as compared with iLIRKO mice (Fig. 6D). An analysis of islet size distribution revealed that the increased beta cell mass observed in iLIRKO IRB mice was due to a significant increase in the proportion of islets of medium size (1000-10,000 µm^2^), whereas the other groups studied showed a quite similar islet size distribution (Fig. 6E). Taken together, these results provide clear evidence that expression of IRA or IRB in the liver is a crucial feature of glucose homeostasis control and, therefore, beta cell mass.

**Fig. 7.**
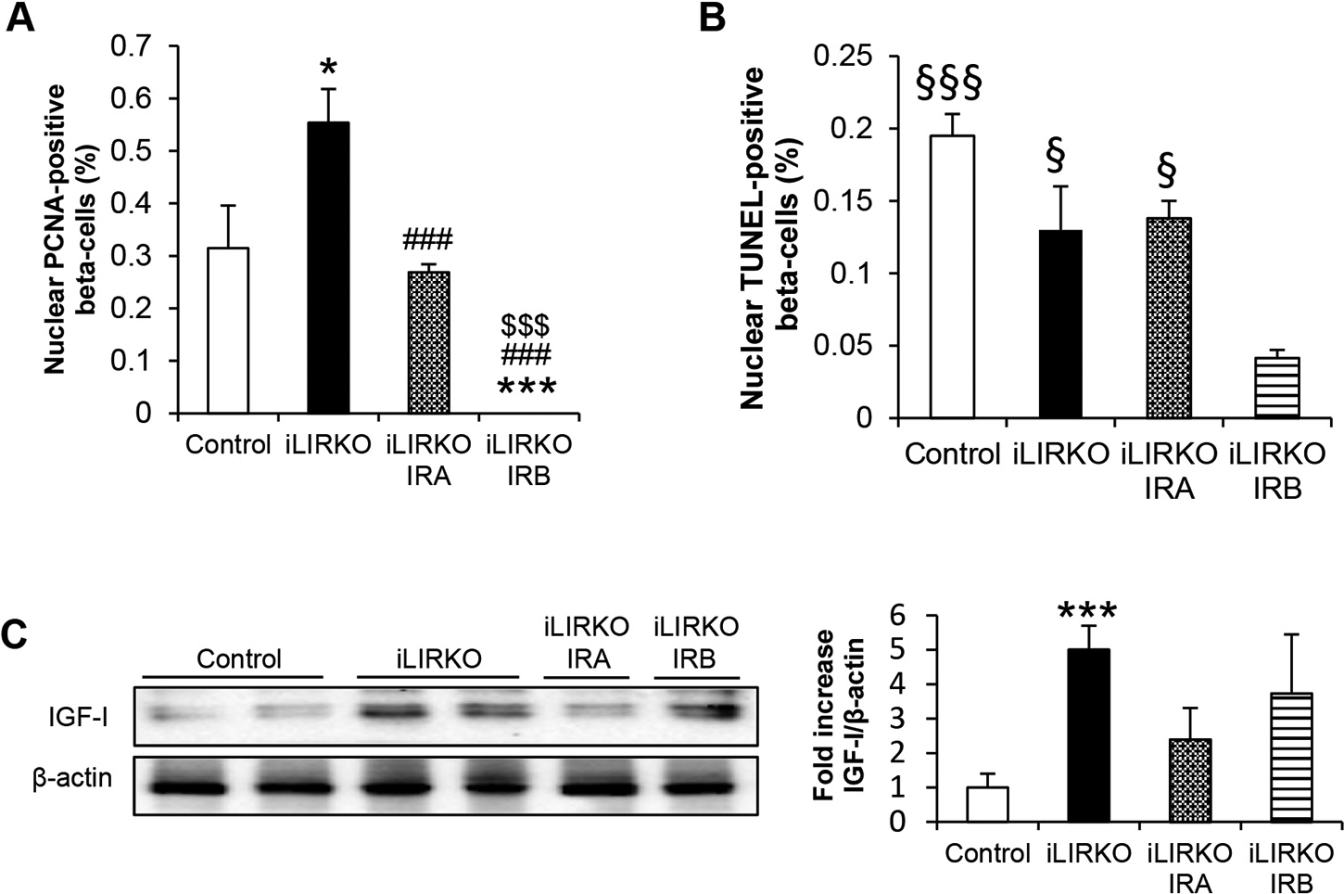
**Hepatic IRA expression reverts beta cell mass expansion by decreasing proliferation and hepatic IGF-I expression.** (A,B) Quantification of nuclear PCNA (A) and TUNEL-positive beta cells (B). (C) Representative IGF-I western blot in liver homogenates from 9-month-old control, iLIRKO, iLIRKO IRA and iLIRKO IRB mice. β-actin was used as loading control. The histogram shows the band intensity quantification. Data are means±s.e.m. for each experimental group (*n*=4). *P<0.05, ***P<0.001 versus control; ^###^*P*<0.001 versus iLIRKO; ^$$$^*P*<0.001 versus iLIRKO IRA; ^§^*P*<0.05, ^§§§^*P*<0.001 versus iLIRKO IRB by unpaired unpaired Student's *t*-test.

To further characterize the mechanisms by which the beta cell hyperplasia reverts in iLIRKO IRA mice we performed two different approaches. First, we stained pancreatic slides with insulin, DAPI and PCNA, a marker of cell proliferation when located in the nucleus (Fig. S1A). Counts of PCNA-positive beta cells revealed significant differences between groups. There was a significant increase in iLIRKO mice compared with control or iLIRKO IRA mice. However, we observed a complete absence of PCNA-positive beta cells in iLIRKO IRB mice, suggesting that the increase in beta cell mass observed in this group could be related to the beta cells being hypertrophic (Fig. 7A). Secondly, we analysed the pancreatic slides by TUNEL to determine the level of apoptosis in each condition (Fig. S1B). Whereas the level of apoptosis was quite similar between control, iLIRKO and iLIRKO IRA mice, we observed a significant decrease in iLIRKO IRB mice as compared with all groups of mice studied (Fig. 7B). As a positive control, we analysed pancreatic slides from neonatal rats, as it has been shown that a transient wave of apoptosis occurs in developing rodent islets between the first and second week of postnatal life (Scaglia et al., 1997; Petrik et al., 1998). These results suggest that the hyperplasia observed in iLIRKO mice is reverted by a decrease in beta cell proliferation rather than an enhanced beta cell apoptosis. As we have previously described, hepatic and circulating IGF-I levels are elevated in insulin-resistant iLIRKO mice, suggesting the presence of a liver–pancreatic endocrine axis (Escribano et al., 2009). Thus, we analysed the hepatic expression of IGF-I in livers from control, iLIRKO, iLIRKO IRA and iLIRKO IRB mice, and found that the increase in IGF-I expression in the liver of iLIRKO mice is markedly downregulated by AAV-IRA treatment. However, in iLIRKO IRB mice, where glucose intolerance remains and the pancreatic beta cell mass continues expanded (Fig. 7C), hepatic IGF-I levels remained elevated. These results suggest a correlation between hepatic IGF-I expression and glucose intolerance. Therefore, these data reinforce our previous results indicating that hepatic IRA, but not IRB, ameliorates glucose intolerance in iLIRKO mice in the long term (Fig. 5B,C). Moreover, regarding safety it is important to emphasize that the marked decrease in hepatic IGF-I expression in iLIRKO IRA mice lessens the possibility of increased IGF-I/IRA signalling in the livers of these animals.

## DISCUSSION

T2DM is a polygenic disease involving polymorphisms in genes encoding proteins that participate in insulin signalling, insulin secretion and intermediary metabolism (Saltiel and Kahn, 2001). Animal models have provided major molecular insights into the role of different cellular pathways involved in the modulation of glucose homeostasis. How and to what extent these genes contribute to the human disease requires further investigation (Neubauer and Kulkarni, 2006).

The liver has the ability to rapidly adapt to blood glucose concentration changes owing to its pivotal role in maintaining glucose homeostasis (El Ouaamari et al., 2013). However, its role in the progression from insulin resistance to fasting hyperglycaemia has been ignored. Previous studies with conditional knockouts and reconstitution models concluded that the progression of insulin resistance to diabetes with fasting hyperglycaemia requires defects in tissues other than liver. Moreover, constitutive deletion of IR in the liver has been reported to impair insulin signalling but does not induce chronic insulin resistance or hyperinsulinemia, probably owing to the hepatic dysfunction developed in these mice (Michael et al., 2000). However, our previous results in iLIRKO mice showed that the inducible liver-specific disruption of IR gives rise to chronic glucose intolerance and hyperinsulinemia without showing any hyperplasic nodules. This mouse model demonstrated that primary hepatic insulin resistance is necessary and sufficient to generate the progressive pathogenesis of T2DM (Escribano et al., 2009).

The unbalanced insulin signalling through compensatory hyperinsulinemia (Malaguarnera and Belfiore, 2011) also characterizes T2DM. It has been shown that in insulin resistant states, insulin loses its ability to suppress glucose production but it largely retains its capacity to drive lipogenesis (Cook et al., 2015). This selective insulin resistance in T2DM has important implications for therapy. It seems preferable to search for new agents that will improve insulin sensitivity in the pathway, leading to suppression of hepatic gluconeogenesis, as well as agents able to enhance glucose uptake in order to decrease the hyperglycaemia (Brown and Goldstein, 2008). In this sense, our group reported *in vitro* that the presence or absence of one or other IR isoform involves changes in the metabolic profile; concisely, that expression of IRA, but not IRB, improves glucose uptake in murine hepatocytes and beta cells by its association with GLUTs (Nevado et al., 2006; Escribano et al., 2015). More recent studies have shown a direct relationship between the metabolic status of individuals with T2DM and the IRA/IRB ratio, suggesting a dynamic regulation of IR splicing depending on the metabolic profile (Besic et al., 2015). As the affinity for insulin is almost identical in both isoforms (Whittaker et al., 2002; Menting et al., 2013) and glucose uptake is insulin-independent in the liver, these changes cannot be related to insulin binding. For all these reasons, it is necessary to dissect the role of each isoform *in vivo* in the liver in a context of T2DM.

In this study, we used iLIRKO mice as a model of T2DM. This model has shown that initial hepatic insulin resistance obtained by insulin receptor deficiency is compensated by a marked hyperinsulinemia, achieved by beta cell hyperplasia. In this regard, we now provide convincing evidence that the increased beta cell mass in response to hepatic insulin resistance previously described in iLIRKO mice (Escribano et al., 2009) was directly related to an increased number of beta cell islets, without changes in their size. Our results demonstrate that global insulin resistance, and therefore glucose homeostasis, can be modified in the long term by expressing IRA, but not IRB, in the liver. Selective expression of IRA was able to restore glucose and insulin tolerance whereas IRB only partially improved insulin sensitivity. Moreover, association between GLUT2 and insulin receptor subunit beta (IRβ) was significantly increased in iLIRKO IRA as compared with iLIRKO IRB. These results agreed with previous *in vitro* studies that showed the same association in IRA-expressing hepatocytes, which was related to an increased glucose uptake (Nevado et al., 2006). Bearing in mind that hyperglycaemia is only importantly decreased in iLIRKO IRA, this could be one of the mechanisms involved in the differential improvement of glucose intolerance. In fact, the favoured association between GLUT2 and IRβ in iLIRKO IRA mice suggests that it is a matter of insulin receptor structure, resulting from the presence or absence of exon 11, instead of structural changes induced upon ligand binding, given the fact that both isoforms present the same affinity for insulin. Moreover, we have found that IRA expression *in vivo* favours insulin signalling on GSK3α/β phosphorylation, inducing an increased glycogen synthesis (data not shown), suggesting that IRA expression not only increases glucose uptake by the hepatocytes, but also favours downstream signalling, leading to an increase in glycogen storage. In addition, we observed a maintained recovery of insulin levels in iLIRKO IRA mice accompanied by a decrease in beta cell mass that reached control values. These results suggest that, as occurs under physiological conditions such as pregnancy where beta cell mass decreases to normal values when insulin resistance remises after delivery, pancreatic beta cell mass adapts to the improvement of hepatic insulin sensitivity by regulating its size (Sorenson and Brelje, 1997; Scaglia et al., 1997; Huang et al., 2009; Rieck and Kaestner, 2010). In the case of IRB-injected mice, beta cell mass and plasma insulin levels were similar to those observed in untreated iLIRKO. These results demonstrate that insulin resistance persists in iLIRKO IRB mice, suggesting a differential role of both isoforms in the regulation of glucose homeostasis.

Because insulin resistance affects all tissues in the body, it is a challenge to identify the signals that promote islet growth in insulin-resistant states. Based on the absence of a detectable increase of beta cell mass in other than the liver-tissue-specific models of insulin resistance (Brüning et al., 1998, 2000; Kulkarni et al., 1999), it is tempting to speculate that the liver controls beta cell mass in insulin-resistant states. In fact, we previously described that different percentages of hepatic insulin receptor deletion correlated with the level of insulin resistance and also with the corresponding increase in beta cell mass, suggesting that the level of hepatic insulin resistance causes a proportional increase in beta cell mass (Escribano et al., 2009). The signals and proteins that mediate islet compensatory response to insulin resistance are currently poorly understood. In this sense, glucose has been reported to promote beta cell growth (Bonner-Weir et al., 1989; Assmann et al., 2009) and is a putative candidate in the induction of beta cell hyperplasia found in iLIRKO mice, which manifest a marked hyperglycaemia. Moreover, we have also described that in iLIRKO mice the expression of hepatic IGF-I is augmented and correlates with an increase in circulating IGF-I levels. In fact, IGF-I levels were correlated to the level of hepatic insulin resistance (Escribano et al., 2009). Thus, our results show that the increased hepatic expression of IGF-I observed in iLIRKO mice decreased in iLIRKO IRA, but not in iLIRKO IRB mice. The lowering of IGF-I lessens the possibility of enhanced activation of the IRA/IGF-I axis that could be related to liver hyperplastic cells. In this sense, although IRA overexpression has been found in tumour samples of hepatocellular carcinoma, it seems that is not a causal factor but a consequence of the activation of other pathways such as EGFR (Chettouh et al., 2013). In any case, as previously mentioned, in this study we precluded IRA overexpression as a caution. In addition, hepatic IGF-I levels are well correlated with the hyperglycaemia, suggesting that both factors could be involved in beta cell growth. Based on these results, it is tempting to speculate that elevated IGF-I plasma levels could become a biomarker of hepatic insulin resistance.

Finally, to further investigate the regression of beta cell mass observed in iLIRKO IRA mice, beta cell proliferation and apoptosis was measured. Adult beta cells proliferate at a very slow rate in basal physiological conditions (Teta et al., 2005; Dhawan et al., 2007; Meier et al., 2008; Perl et al., 2010). However, in a pathological situation like the insulin resistance observed in iLIRKO mice, the proliferation rate was increased. Our results show that hepatic IRA expression was able to reduce islet mass through decreasing beta cell proliferation rate to control values, without significant alterations in pancreatic beta cell apoptosis.

Taken together, the results in this study highlight the central and complex role played by hepatic insulin receptor isoforms in the control of glucose metabolism. Based on these findings, *in vivo* long-term AAV-mediated hepatic expression of IRA, but not IRB, could act as a glucose uptake promoter regulating hyperglycaemia, improving glucose homeostasis, precluding beta cell mass expansion and, therefore, avoiding the final beta cell failure.

## MATERIALS AND METHODS

### Mice and diets

*Insr^lox/lo^_x_* [hereafter IR^(lox/lox)^] mice were created by homologous recombination using an insulin receptor gene-targeting vector with loxP sites flanking exon 4 (Brüning et al., 1998). For liver-specific deletion we used transgenic mice with the tamoxifen-dependent Cre-ER^T2^ recombinase coding sequence under control of the albumin promoter (Schuler et al., 2004). The iLIRKO mice were generated by crossing C57Bl/6 IR^(lox/lox)^ and heterozygous C57Bl/6 Alb-Cre-ER^T2^; littermate IR^(lox/lox)^ were used as control. Genotyping of the IR^(lox/lox)^ and Alb-Cre-ER^T2^ transgenic mice was performed by PCR using genomic DNA isolated from the tip of the tail of 3- to 4-week-old mice as previously described (Schuler et al., 2004; Escribano et al., 2009). After weaning, iLIRKO and control mice [IR^(lox/lox)^] were fed with a soy-free diet (RMS-0909-US-EN-02-DS-2016; Harlan Teklad, Barcelona, Spain) for two weeks followed by two weeks of tamoxifen diet (TD.09327, Harlan) in order to induce translocation of Cre to the nucleus (Schuler et al., 2004). Following this, animals were fed with a standard chow *ad libitum*. Only male animals were studied and maintained on a 12-h light-dark cycle. All animal experimentation was conducted in accordance with the accepted standards of animal use approved by the Complutense University of Madrid Committee.

### Viral constructs and vector production and purification

Recombinant AAV vectors were constructed with a transgene cassette coding sequence for the individual spliced single chain isoforms of *INSR* either containing or lacking exon 11 (IRB and IRA, respectively), or the reporter *luc* under the regulation of a liver-specific promoter, *α1-antitrypsin* (*AAT*). Coding sequences for the human IR isoforms were a generous gift of C.R. Kahn (Joslin Diabetes Center, Boston, MA). The transgene cassette was flanked by AAV2 wild-type inverted terminal repeats. rAAV8 vectors were produced as previously described (Gil-Farina et al., 2013). Viral titres were determined by qPCR, performed three times in triplicate at three different dilutions.

### AAV administration

5-month-old mice were injected intravenously with the AAVs. For all procedures, animals were anaesthetized by intraperitoneal (IP) injection of a mixture of xylacine (Rompun 2%, Bayer, Leverkusen, Germany) and ketamin (Imalgene 50, Merial, Lyon, France) at 1: 9 v/v.

### Immunoprecipitation and western blot

Tissues were homogenized as described (Brüning et al., 1998). Western blot analyses of insulin signalling proteins were performed on liver homogenates as previously described (Valverde et al., 1998). For immunoprecipitation, after protein content determination, equal amounts of protein (400 μg) were immunoprecipitated overnight at 4°C with anti-GLUT-2 antibodies (C-19) (1:500; sc-7580, Santa Cruz Biotechnology, Dallas, TX). The immune complexes were collected on protein A-agarose beads and submitted to SDS-PAGE. For western blot, the antibodies used were anti-insulin receptor β subunit (1:1000; sc-711), anti-GLUT-2 (H-67) (1:500; sc-9117) and anti-IGF-I (1:200; sc-9013) (all from Santa Cruz Biotechnology) and anti-β-actin (1:5000; A2228, Sigma-Aldrich, St. Louis, MO). Rabbit and mouse primary antibodies were immunodetected using horseradish peroxidase-conjugated polyclonal anti-rabbit or mouse antibodies, respectively (1:5000; NA931V and NA934V, GE Healthcare, Buckinghamshire, UK). Loading was normalized by β-actin. The band intensities were quantified using ImageJ software (http://rsb.info.nih.gov/ij).

### Metabolic tests

Glucose tolerance tests (GTT) were performed by intraperitoneal administration of 2 g/kg body weight of glucose after a 16 h overnight fast. Blood glucose was monitored using Accu-Check blood glucose strips and glucometer (Roche, Penzberg, Germany). Insulin tolerance tests (ITT) were performed in the random-fed state at 11:00. Animals were injected with 1 U/kg body weight of human regular insulin (Humulin regular, Eli Lilly, Indianapolis, IN) and blood glucose levels were measured at indicated times. ITT data are presented as percentage of initial blood glucose concentration. Insulin ELISA (Millipore, Billerica, MA) was performed with plasma samples obtained from 16 h overnight fasted mice. Total cholesterol kit (Wako, Saitama, Japan) and total triglycerides kit (Thermo Fisher, Waltham, MA) were used with plasma and liver samples obtained from mice fasted for 24 h followed by 1 h of refeeding. Hepatic lipids were isolated as described (Folch et al., 1957).

### mRNA and genomic DNA expression

Liver RNA was prepared using Trizol (Life Technologies, Carlsbad, CA), cDNA was synthesized using High Capacity reaction kit (Applied Biosystems, Carlsbad, CA) and PCR was performed with DNA AmpliGel Master Mix (Biotools, Madrid, Spain). Genomic DNA from liver was extracted with DNA purification system (Promega, Madison, WI) and qPCR was performed using FastStart Universal SYBR green master mix (Roche) using the forward primer (intron 3) 5′-GTCCGCTTGTCCCACCAG-3′ and reverse primer (exon 4) 5′-CAATGGTCTTCTCACCTTCG-3′. The primers used to determine the deletion of the exon 4 were 5′-CTGTTCGGAACCTGATGAC-3′ and 5′-ATACCAGAGCATAGGAG-3′. Primers flanking the human exon 11 were 5′-AGGAAGACGTTTGAGGATT-3′ and 5′-CACCGTCACATTCCCAACAT-3′. A 316 bp band corresponds to the IRB isoform and 280 bp to the IRA isoform.

### Immunohistochemistry, immunofluorescence, islet morphometry and analysis of cell proliferation

Tissue samples were fixed overnight in 4% formaldehyde made up in 10% phosphate-buffered saline, and routinely paraffin-embedded. Each liver block was serially sectioned (7 μm) and haematoxylin and eosin (H&E)-stained using standard techniques. For GFP staining, we used anti-GFP antibodies (B-2) (1:100; sc-8334, Santa Cruz Biotechnology), and the corresponding negative controls were performed in the same samples without anti-GFP primary antibodies. Total GFP-positive and GFP-negative cell numbers per section were counted to evaluate the transduction percentage. At least 1500 hepatocytes were counted per liver.

Each pancreatic or hepatic block was then serially sectioned (5 μm) throughout its length to avoid any bias owing to regional changes in islet distribution and islet cell composition, and was mounted on slides. Sections at fixed intervals throughout the block (every 72nd section) were incubated with guinea pig antibodies against insulin (1:100; A0564, Abcam, Cambridge, UK) or Ki-67 (1:200; M7240, Dako, Glostrup, Denmark). Visualization of immunocomplexes was carried out using appropriate secondary antibodies (1:200; ab-6771, Abcam and 1:200; P0447, Dako) and a diaminobenzidine substrate kit for peroxidase (Agilent Technologies, Santa Clara, CA). Beta cell fractional area was determined by calculating the ratio between the area occupied by insulin-positive cells and the area occupied by total pancreatic cells. The total islet number per section (mm^2^ of pancreatic tissue) was counted, and islets were arbitrarily classified by their area (μm^2^) to evaluate the distribution of islet sizes.

Proliferating beta cells were identified through PCNA nucleus location by co-staining for PCNA (F-2) (1:100; sc-56, Santa Cruz Biotechnology), DAPI and insulin. Beta cell replication rate was expressed as the percentage of PCNA-positive beta cells. At least 1500 beta cells were counted per pancreas. Beta cell apoptosis was estimated by TUNEL assay (ApopTag Peroxidase In Situ Apoptosis Detection Kit, Millipore) coupled to insulin staining as previously described (Miguel-Santos et al., 2010) and the beta cell apoptosis rate expressed as the percentage of apoptotic-positive beta cells. Images of stained sections were acquired using a digital camera (XCD-U100CR, Sony, Tokyo, Japan) attached to a light microscope (Eclipse 8i, Nikon, Tokyo, Japan). Morphometric analyses were performed with automated image analysis software (Histolab, Microvision Instruments, Gothenburg, Sweden).

### Bioluminescence imaging

Mice were immobilized with intraperitoneal anaesthesia (a mixture of xylacine and ketamine). The substrate luciferin (150 µg/kg dissolved in phosphate-buffered saline; Promega, Madison, WI) was intraperitoneally injected. After 10 min, animals were placed in the dark chamber for light acquisition in an IVIS charge-coupled device camera system (Xenogen, Alameda, CA) and analysed with the Living Image 2.20 software package (Xenogen). A region of interest covering the whole animal was defined and quantification of light emission was performed in photons/second. Time exposure ranged from 1 s to 5 min depending on light intensity.

### Statistical analysis

Data are presented as means±s.e.m. from at least three independent experiments. Regarding *in vivo* experiments we used at least four mice. Differences between two groups were assessed using unpaired two-tailed *t*-tests. Data involving more than two groups were assessed by analysis of variance (ANOVA) with Bonferroni post test. A *P*-value of <0.05 was considered statistically significant.
